# A Short Commentary on Non-specialist-mediated Interventions for Children with Autism Spectrum Disorder

**DOI:** 10.7759/cureus.4831

**Published:** 2019-06-04

**Authors:** Tayyaba Afzaal, Ahmed Waqas, Sadiq Naveed

**Affiliations:** 1 Psychology, Government College University, Lahore, Pakistan; 2 Psychiatry, Combined Military Hospital Lahore Medical College and Dental College, Lahore, PAK; 3 Psychiatry, University of Kansas Medical Center, Kansas City, USA

**Keywords:** autism, interventions, non-specialist, peers, parent, teachers

## Abstract

Autism spectrum disorder (ASD) is a lifelong neurodevelopmental disability delineated by social and communication impairments, restricted interests and repetitive behaviors, and sensory hypo- or hyper reactivity to sensory inputs. Early recognition and timely intervention are essential for individuals with ASD and the long-term prognosis for these individuals. The specialist-mediated interventions can be expensive for patients, their families, and the healthcare system. It is important to provide a naturalistic intervention, especially in the context of cost issues, the impact of early intervention of prognosis, limited resources in developing countries, lack of healthcare providers, and healthcare disparities. The current article reviews non-specialist-mediated interventions of children and adolescents with ASD.

## Introduction and background

Autism has traditionally been viewed as a severe but rare disorder for which little can be done. Autism spectrum disorder (ASD) is a lifelong neurodevelopmental disability delineated by social and communication impairments, restricted interests and repetitive behaviors, and sensory hypo- or hyperreactivity to sensory inputs. There has been an increase in the diagnosis of ASD by almost 150% in the last two decades. The prevalence of ASD is estimated to be around 14.5 per 1000 (one in 69) among children with age eight years. It is more prevalent in boys than in girls [1]. This increase is possibly secondary to increased education and awareness, better diagnostic tools, and an increased effort to early diagnosis of these children.

Early recognition and intervention can improve the treatment outcomes and prognosis for individuals with ASD. It is significantly important in the context of healthcare costs related to ASD and related comorbidities. The lifetime cost of services for an individual with an ASD and intellectual disability is reported to be approximately $2.4 million in the United States and £1.5 million in the United Kingdom (UK). This cost is lesser in individuals with ASD without intellectual disability and reported to be around $1.4 million in the United States and £0.92 million ($1.4 million) in the UK. The higher financial expenditure in individuals with comorbid ASD and ID indicates a higher needs for these children [2]. 

Considering the cost issues and the impact of early intervention of prognosis, it is important to provide naturalistic intervention. This becomes critical in the backdrop of limited resources in developing countries, lack of healthcare providers, and healthcare disparities. Many children with ASD belonging to developing countries failed to achieve their full potential. The poor prognosis, decreased productivity, and higher healthcare costs pose a significant challenge for the healthcare system and stakeholders. There has been an increased focus on using the support system of an individual with ASD to provide targeted interventions in recent years [3]. These support systems can involve parents, family members, teachers, siblings, and peers.

This article reviews the evidence-based non-specialist-meditated interventions for children and adolescent with ASD. This treatment approach has been found to be very cost-effective, for instance, the Play and Language for Autistic Youngsters (PLAY) project, costs only $3500 to $4500 per year per child as compared to $40,000 to $80,000 per year for interventions delivered by mental health professionals [4].

## Review

Early interventions sketched for ASD require more skilled training and proved ineffective when administered by the parents of young children. Therefore, contemporary studies purposed that pre-requisite of assistance by non-specialist providers in school and community settings may comprise an effective substitute, including behavior modification approaches and parent-mediated interventions. Recent researches come up with non-specialist mediated interventions designed for children with ASD in the United States [5]. These programs are designed for different settings such as home or school and differ in their therapeutic strategies, focus on different symptoms. We explained some of these incredible non-specialist mediated interventions for individuals with ASD below.

Adaptive response teaching

A recent study by Baranek and colleagues was designed to focus on the usefulness of enlisting 12 months at risk of ASD. This intervention leads to describing parents about the utilization of community early intervention services and assessing the potential of preliminary efficacy of a parent-mediated intervention. The main objectives of the study were to target key domains like social communication (social play, joint activity, vocalization) and sensory regulation (self-regulation, attention and arousal, exploration). Adaptive response teaching inventory shows more beneficial effects particularly during the active phase of treatment. The analysis illustrates that the parent-mediated intervention improved the outcomes for the core symptoms of ASD, suggesting a need for earlier access to these individuals in their community [6].

Social-emotional neuroscience endocrinology (SENSE) theatre

The Social-Emotional NeuroScience Endocrinology (SENSE) theatre by Corbett and colleagues used a unique strategy to address the social and emotional issues of youth with autism. The prime focus of the study was to engage children with ASD (age: 8-17 years) with typically developing children who were specially trained to serve as models for social and communications skills that are the core symptoms of ASD. The study examined the influence of the intervention on reducing anxiety and stress. The theatre uses procedures such as role-play, character building, making it up as go along and completing in public appearances of a play. These promising findings suggest that theatre-based intervention can result in improved social competence, by addressing social-cognitive functioning and children with autism can learn with unconventional techniques in unique settings [7].

Parent-mediated cognitive behavioral therapy (CBT)

Parent-mediated cognitive behavior intervention concentrates on educating parent to recognize the clinical signs of anxiety in preschool children and apply cognitive behavioral therapy (CBT)-based coping strategies to manage these complex issues. In promising research, Cook and colleagues examined the effectiveness of parent-mediated CBT and analyzed the connection between thoughts, feelings, and behaviors in the context of early childhood development and care anxiety. Parents first received coaching in CBT skills and strategies which they, in turn, can easily interpret to the children. The intervention addresses the key domains comprising parent skills training in emotion regulation, psychoeducation, construction of an exposure hierarchy, graded exposure, and effective education [8].

Parent-mediated communication-focused treatment

In a study, Green and colleagues assessed the influence of the communication style of parents on the social development and communication of children with autism. The Parent-mediated Communication-focused Treatment (PACT) involves parents and child one to one meeting with the therapist. The target of the intervention was to constrain parents’ responsiveness towards child communication through video feedback method. The tactics like action routines, familiar repetitive language, and pauses were used to enhance incremental development of child communication [9].

Telehealth parent-mediated intervention

The technology already has significant impacts on health services. The Improving Parents as Communication Teachers (ImPACT) Online, a telehealth program, can be a cost-effective option with a possibility of maximizing the number of hours a child receives. It proved to help in qualifying parents to implement telemedicine services themselves and has the potential to eliminate traditional models of services. Telehealth interventions allow parents to engage independently with the interactive program or also can acquire assistance from the therapist. The website includes self-directed lessons, slideshows, self-check questions, exercises, and video clips. Parents were encouraged to complete the interventions with their children in an endeavor to improve their communication skills. However, sessions assisted by therapist group showed exceptional outcomes in their use of the intervention [10].

Joint attention, symbolic play, engagement, & regulation (JASPER)

Joint Attention, Symbolic Play, Engagement, & Regulation (JASPER) revealed the main segments of the intervention in its name which holds joint attention, symbolic play, engagement, and regulation. Dr. Connie Kasari developed a novel strategy to encourage parents and therapists using naturalistic approaches by following the lead of children considering families’ daily life routines. The primary goal of the intervention is to promote dyadic mutual engagement of parent and child by directly involving in an activity together, such as playing with toys. When the dyad of parents and children parents has similar goals during play, it creates more opportunities for adults to move, sound and appears similar to the child with whom he is communicating and permits the adult to enter the child’s domain by adjusting the focus, the needs, and interest of the children [11].

Qigong sensory treatment

Qigong sensory training is a whole body massage etiquette, which is based on Chinese medicine. The massage is done by tapping from the head towards the hands and feet-watches the blood flow direction and shifts oxygenated blood to the skin and sensory nerves. According to Chinese medicine blockage, in the sensory channels affects the way of taking information from the environment in children with autism. Hence, the treatment uses a massage technique in improving sensory and self-regulatory impairment by boosting circulation to the skin. This therapy resulted in a calmer child and better social skills in treated children and suggested better outcomes for behavior and communication-related measures of autism [12].

Play and language for autistic youngsters (PLAY) project

A new intervention, that is evidence-based, named as the Play and Language for Autistic Youngsters (PLAY) project has been developed by Dr. Richard Solomon. The PLAY project is executed by parents, using their relationship and knowledge of the children, in a natural home environment followed up by the office and clinic visits. The key features of intervention include video recording, coaching, modeling, and written feedback. Children with ASD have a lot of potentials; unlocking their potential requires time and engagement through play sessions and daily activities. The PLAY project intervention engages the children in the play and helps with his or her development. The project allows a more cost-effective alternative to traditional therapeutic intervention [4].

Peer-mediated intervention

This research by Roeyers focuses on the impact of social intercommunication of typically developing children with ASD. At first, children without ASD received training and were instructed about modeling appropriate behavior skills (e.g., how to counter in a viable aggressive situation). The study was conducted in the naturalistic environment under the supervision of adults without interloping during the play sessions. The playrooms were well equipped with toys such as balls, bowling pins, puppets, cars and so forth [13].

Parent-mediated intervention for autism spectrum disorder (PASS)

The Parent-mediated Intervention for Autism Spectrum Disorder (PASS) is a parent-mediated intervention assessed in South Asia (Pakistan & India) to see the practicability and acceptability. A video feedback method was used to direct the simultaneous response of parents to child communication and subdue over-directive rejoinders. The novel elements of the intervention were that it includes simplification of the language according to the cultural context, delivery by lay health workers in the homes of families, cost-effectiveness, and inclusion of all family members besides parents [14].

Joint attention-mediated learning (JAML)

Joint attention is a pivotal social communication skill often absent or impaired in young children with the ASD. The Joint Attention-mediated Learning (JAML), a parent-led intervention, mainly concentrates on enhancing joint attention. JAML is based on the notion that a parent can largely take apart to grow and enhance their child's language, social, and cognitive development. In focusing on faces and turn-taking phases, children are taught to look at parent followed by the engagement in repetitive play with children and parents that acknowledges the other’s shared interest by accommodating the parent’s turn. Finally, triadic engagement involves the use of toys in the joint attention phase [15].

Learning experiences and alternative program for preschoolers and their parents (LEAP)

Learning Experiences and Alternative Program for Preschoolers and Their Parents (LEAP) is an evidence-based intervention for the young children with ASD and the only model which is executed in public schools. In this paradigm, a small group of typically developing children is trained and taught with a small number of children with ASD. LEAP consists of unique features such as integrated preschool program, skills training for parents and pivotal response training and reinforced by age-appropriate rewards and praise for demonstrating correct actions and behaviors. Long-term effects are currently being appraised [16]. 

Hanen’s ‘more than words’ (HMTW) 

Hanen’s ‘More than Words’ (HMTW) is an absolute family-centered training program, to build parents skills to develop a child’s communication skills. HMTW is based on the ‘social-pragmatic’ theory of language development with practical and powerful strategies to help the child to communicate and interact during everyday routines and activities in a naturalistic setting. This approach modifies the environment, adapts the interactions of others and builds functional skills with a person-centered approach around interests and goals. The intervention consists of one to one interaction with parents and child, group sessions and video-based reinforcement [17].

Peer-mediated intervention

In a study, Kasari et al. investigated the effects of teaching social skills by typical peers to the children with ASD in general education settings. The peer-mediated intervention serves the purpose to develop more meaningful interaction in children with ASD via giving training to typical peers, modeling and role-playing. They were also explained how to identify appropriate and inappropriate behaviors on the playground, tactics for boosting positive social interactions and initiate play interactions. Both groups of peer were reinforced for appropriate engagement [18].

Focused playtime intervention (FPI)

Focused Playtime Intervention (FPI) is a parent-mediated intervention with a goal to improve responsive parental communication. FPI builds on the idea to educate parents that involve 12 in-home training sessions (one session per week for 12 weeks, 90 min per session). The intervention incorporates two phases: the first phase provides plentiful opportunities for parent and interventionist to engage in reciprocal interactions between the dyad of children and parents. The second phase entails only parents (possibly siblings too) and child play with each other for some time through video feedback, conventional teaching, and emphasis on homework assignments [19].

Discussion

This article provides an opportunity to review different interventions targeting social and communication skills and restricted interests in children and adolescents with ASD. These interventions use the support system of a child with ASD. It serves several purposes including intervention delivered by familiar people in the daily intervention of child with minimal resources allocation in the backdrop of a child struggling with social and communications skills [20]. A systematic review and meta-analysis took a deeper dig into the quantitative and quantitative efficacy of these interventions [3]. This analysis suggested favorable evidence of these interventions in targeting parent-child relationship and core symptoms of ASD. In a country like Pakistan, resource limitation is a critical issue considering the lack of psychologist, child psychiatrists, occupational therapists, and school counselors. The authors call for the attention of policymakers, healthcare leaders, stakeholders, and advocates to provide a framework where these interventions are readily available to children with ASD [20].

## Conclusions

The non-specialist-mediated intervention has a favorable efficacy for the core symptoms of autism. Lack of access to healthcare and affordability are potential limitations in providing timely interventions. These interventions using a support system of a child may help in the impact on public health.
